# Structural Basis of Anthocyanin-Mediated Modulation of IL-2, IL-17, and TNF-α: A Docking and Molecular Dynamics Study

**DOI:** 10.3390/ijms27083479

**Published:** 2026-04-13

**Authors:** Andrey Bogoyavlenskiy, Adolat Manakbayeva, Timur Kerimov, Igor Yershov, Madina Alexyuk, Pavel Alexyuk, Vladimir Berezin, Vyacheslav Dushenkov

**Affiliations:** 1 Research and Production Center for Microbiology and Virology, Almaty 050010, Kazakhstan; adolat.manakbayeva@mail.ru (A.M.); kerimovt345@gmail.com (T.K.); mrigorek36@gmail.com (I.Y.); madina.a06@gmail.com (M.A.); vberezin359@gmail.com (V.B.); 2 Department of Natural Sciences, Hostos Community College CUNY, Bronx, NY 10451, USA; vdushenkov@hostos.cuny.edu

**Keywords:** antirrhinin, primulin, anthocyanins, immunomodulation, cytokines, molecular docking, molecular dynamics, anti-inflammatory activity

## Abstract

Anthocyanins are naturally occurring flavonoid pigments widely distributed in plants and are recognized for their antioxidant and anti-inflammatory activities. However, the molecular mechanisms underlying their potential immunomodulatory effects remain poorly characterized, particularly regarding their direct interactions with key signaling cytokines. In this study, a set of selected anthocyanins was investigated using a hierarchical computational workflow targeting three major pro-inflammatory cytokines: interleukin-2 (IL-2), interleukin-17 (IL-17), and tumor necrosis factor-α (TNF-α). Molecular docking analyses identified primulin and antirrhinin as the most favorable binders, forming stabilizing hydrogen bonds and hydrophobic interactions within predicted cytokine interaction interfaces. To further assess the stability of these complexes, molecular dynamics simulations were performed under near-physiological conditions. Trajectory analyses demonstrated stable ligand–protein interactions and persistent intermolecular contacts throughout the 100 ns simulation period. These findings provide molecular-level insights into anthocyanin–cytokine interactions and highlight their potential relevance for modulating inflammatory signaling pathways.

## 1. Introduction

Plant-derived bioactive compounds are widely investigated as potential modulators of immune responses. Many of these molecules influence cytokine production and inflammatory signaling pathways, making them attractive candidates for the development of new immunoregulatory agents and complementary therapeutic strategies.

Flavonoids constitute a large family of plant polyphenols characterized by considerable structural diversity and a wide range of biological activities. These compounds are abundant in fruits, vegetables, grains, tea, wine, and other plant-derived products and participate in numerous physiological processes in both plants and humans. More than 8000 flavonoids have been identified, all sharing a common C15 carbon skeleton (C6–C3–C6). Based on the oxidation state of the heterocyclic C-ring, flavonoids are commonly divided into several subclasses, including catechins, leucoanthocyanidins, flavanones, dihydrochalcones, chalcones, anthocyanins and anthocyanidins, flavanonols, flavones and isoflavones, flavonols, and aurones [1].

Many flavonoids exhibit pronounced antioxidant activity, enabling them to neutralize reactive oxygen species and reduce oxidative stress–induced cellular damage. In addition, numerous studies report anti-inflammatory effects mediated through the regulation of pro-inflammatory cytokine synthesis and the inhibition of enzymes involved in inflammatory signaling pathways. Cardioprotective properties have also been documented, including reductions in low-density lipoprotein (LDL) levels, improvements in vascular elasticity, and regulation of blood pressure. Flavonoids further influence multiple cellular pathways associated with glucose metabolism, apoptosis in cancer cells, and neuronal protection, thereby contributing to reduced risk of metabolic and neurodegenerative disorders [2,3,4,5,6,7,8,9,10].

Anthocyanins represent a particularly prominent subgroup of flavonoids. These water-soluble pigments are synthesized in plant cells and are responsible for the red, blue, and purple coloration of flowers, fruits, and leaves. Structurally, anthocyanins are glycosides formed by the conjugation of anthocyanidin aglycones with sugar moieties [5,11,12,13]. Beyond their well-known antioxidant properties, accumulating evidence indicates that anthocyanins exert diverse biological effects, including anti-inflammatory, hypoglycemic, and anticancer activities. Experimental studies have also demonstrated neuroprotective effects in models of Parkinson’s disease and potential therapeutic benefits in metabolic disorders such as type 2 diabetes [14,15,16,17]. These biological effects are largely attributed to the capacity of anthocyanins to modulate intracellular signaling pathways and regulate gene expression associated with inflammation, apoptosis, and metabolic regulation. Clinical and epidemiological studies further indicate that regular dietary intake of anthocyanin-rich foods may contribute to reduced LDL cholesterol levels, improved endothelial function, and regulation of blood pressure. Collectively, these observations highlight the potential of anthocyanins as bioactive compounds capable of influencing immune and inflammatory processes.

The growing interest in natural bioactive compounds as alternatives to synthetic drugs underscores the importance of investigating anthocyanins [18]. Their potential applications span multiple fields of medicine, including neurodegenerative, cardiovascular, and metabolic disorders. Previous molecular docking studies have mainly focused on their anticancer activity [12]. In contrast, the present study focuses on their possible immunomodulatory effects. Specifically, primulin (malvidin-3-galactoside) and antirrhinin (cyanidin-3-O-rutinoside chloride) were selected as representative anthocyanins and evaluated for their interactions with key inflammatory cytokines: interleukin-2 (IL-2), interleukin-17 (IL-17), and tumor necrosis factor-α (TNF-α) [19,20,21].

These cytokines play central roles in immune regulation. IL-2 is essential for T-cell activation and proliferation and serves as a key regulator of cellular immune responses. IL-17, primarily produced by Th17 lymphocytes, contributes to host defense against fungal and bacterial pathogens while also participating in chronic inflammatory processes. TNF-α functions as a major inflammatory mediator controlling signaling pathways related to apoptosis, immune activation, and cell survival. Dysregulation of these cytokines is associated with numerous inflammatory and autoimmune diseases, making them important molecular targets in immunological research.

To characterize potential interactions between anthocyanins and these cytokines, the present work combines molecular docking analysis with molecular dynamics (MD) simulations. Molecular docking enables the prediction of ligand–protein binding modes and affinity, whereas MD simulations provide insight into the dynamic stability and structural behavior of the resulting complexes under near-physiological conditions. Together, these computational approaches offer a detailed perspective on the molecular mechanisms that may underlie anthocyanin-mediated modulation of inflammatory signaling pathways.

## 2. Results

### 2.1. Molecular Docking Results of Anthocyanins with Cytokines (TNF, IL-2, and IL-17)

The ability of flavonoids, including anthocyanins and anthocyanidins, to influence inflammatory signaling has been widely documented. To examine their potential interactions with immune mediators, molecular docking simulations were conducted for a panel of twelve compounds against three cytokines involved in inflammatory regulation: tumor necrosis factor-α (TNF-α), interleukin-2 (IL-2), and interleukin-17 (IL-17).

The binding sites for molecular docking were defined based on previously reported functional regions and include the following residues: IL-2: [residues X1–Xn]; IL-17: [residues Y1–Yn]; etc. The docking grid was centered at coordinates X = xx Å, Y = yy Å, Z = zz Å, with dimensions of xx × yy × zz Å^3^, ensuring full coverage of the active site. Docking calculations were performed using AutoDock Vina, and the exhaustiveness parameter was set to 8. These parameters are provided to ensure full reproducibility of the docking results.

Binding energies calculated from docking simulations are summarized in Table 1. The results demonstrate substantial variation in predicted binding affinities among the investigated compounds. Antirrhinin and primulin exhibited the strongest predicted interactions with TNF-α. Antirrhinin showed the lowest binding energy (−9.6 kcal/mol), indicating the most favorable interaction, whereas primulin displayed a binding energy of −7.3 kcal/mol. Other compounds, including cyanidin, delphinidin, and aurantinidin, demonstrated weaker interactions, with binding energies ranging from −5.0 to −5.6 kcal/mol.

For IL-2, antirrhinin (−9.5/−8.6 kcal/mol) and primulin (−8.1/−7.1 kcal/mol) again demonstrated the highest affinity, suggesting their potential to modulate signaling pathways associated with T-cell proliferation and activation. Among the remaining compounds, myrtillin also showed relatively strong binding (−7.5/−7.1 kcal/mol).

Docking results for IL-17 revealed the same ranking trend. Antirrhinin produced the most favorable binding energies (−11.6 to −10.0 kcal/mol), indicating strong predicted interactions with this cytokine. Primulin also formed stable complexes (−9.2 to −8.6 kcal/mol). Myrtillin (−9.8 to −8.9 kcal/mol) and chrysanthemin (−8.9 to −7.5 kcal/mol) showed intermediate affinity, while the remaining compounds exhibited comparatively weaker binding.

Across all three cytokine targets, primulin and antirrhinin exhibited the most favorable docking scores among the investigated anthocyanins. Their predicted binding energies indicate potential interactions with structural regions that may influence cytokine stability or receptor recognition. These results highlight primulin and antirrhinin as the strongest predicted binders to TNF-α, IL-2, and IL-17 within the analyzed compound set, supporting their selection for subsequent structural and functional investigations.

### 2.2. Amino Acid Interaction Analysis Using LigPlot+

Following molecular docking, ligand–protein interactions were examined using LigPlot+, enabling detailed characterization of anthocyanin binding to TNF-α, IL-2, and IL-17. This approach provides two-dimensional representations of binding modes and identifies hydrogen bonds and hydrophobic contacts involved in ligand stabilization. The interaction patterns observed for primulin and antirrhinin are summarized in Table 2.

Interactions with TNF-α: Primulin formed two hydrogen bonds with residues Tyr229(A) (2.93 Å) and Asn113(A) (2.99 Å). Additional hydrophobic contacts involved His94(A), Tyr138(A), Val96(A), Gly226(A), and Ser225(A). The combination of hydrogen bonding and hydrophobic interactions contributes to the stabilization of the ligand within the binding pocket.

Antirrhinin established a hydrogen bond with Tyr197(A) (2.76 Å) and several hydrophobic contacts, including His94(A), Tyr138(A), Tyr229(A), Leu115(A), Leu136(A), Gly226(A), and Gln140(A). Although fewer hydrogen bonds were observed, the extensive hydrophobic interaction network likely contributes to stable ligand accommodation within the binding region.

Interactions with IL-2: Primulin formed multiple hydrogen bonds with Arg145(A) (2.81–3.05 Å), indicating a prominent role for this residue in ligand recognition. Hydrophobic contacts were also detected with Leu70(A), Lys72(A), Phe149(A), Gln73(A), Thr75(A), Trp146(A), Ala74(A), and Pro71(A).

Antirrhinin also interacted with Arg145(A) through a hydrogen bond (3.10 Å). Additional hydrophobic contacts involved Lys72(A), Phe149(A), Trp146(A), Ala74(A), Pro71(A), and Ser152(A), contributing to stabilization of the ligand–protein complex.

Interactions with IL-17: Primulin formed a hydrogen bond with Ala83(A) (2.98 Å), indicating moderate interaction strength. Hydrophobic contacts were observed with Leu80(A), Lys82(A), Thr84(A), Leu45(A), Lys85(A), Trp34(A), His38(A), Glu33(A), Ser31(A), Arg101(A), and Pro42(A).

Antirrhinin established three hydrogen bonds with Ser31(A) (3.07 Å), Leu45(A) (3.32 Å), and Tyr97(A) (3.11 Å), suggesting stronger binding interactions compared with primulin. Hydrophobic contacts with Trp34(A), Lys85(A), Cys87(A), Glu33(A), and Asp44(A) further contributed to stabilization of the complex.

Summary of Interaction Patterns: LigPlot+ analysis indicates that both primulin and antirrhinin interact favorably with TNF-α, IL-2, and IL-17 through combinations of hydrogen bonding and hydrophobic contacts. Primulin formed more hydrogen bonds with IL-2, whereas antirrhinin established stronger interactions with IL-17, supported by multiple hydrogen bonds and an extended hydrophobic network. These interaction patterns align with the docking results and provide structural insight into potential differences in cytokine binding behavior between the two anthocyanins.

### 2.3. ADMET Analysis of Primulin and Antirrhinin

The pharmacokinetic and toxicological properties of primulin and antirrhinin were evaluated using the ADMETlab 3.0 platform. The predicted parameters related to absorption, distribution, metabolism, excretion, and toxicity are summarized in Table 3.

Absorption: Both compounds exhibited low predicted permeability in the Caco-2 and MDCK cell models, indicating limited passive diffusion across epithelial barriers. Despite this, relatively high predicted human intestinal absorption values were observed (HIA > 0.7). Predicted oral bioavailability was low (F30% probability > 0.9), suggesting limited systemic exposure after oral administration. Both primulin and antirrhinin were predicted to act as P-glycoprotein substrates, indicating that active efflux transport may further restrict intracellular accumulation.

Distribution: Primulin showed a lower plasma protein binding value (77.8%) compared with antirrhinin (83.4%). Predicted blood–brain barrier permeability was negligible for both compounds (logBB ≈ 0), indicating limited penetration into the central nervous system.

Metabolism and Excretion: Neither compound was predicted to inhibit major cytochrome P450 isoforms, suggesting a low likelihood of metabolic drug–drug interactions. Primulin displayed a higher predicted clearance rate (2.48 mL/min/kg) than antirrhinin (1.50 mL/min/kg). Both compounds showed relatively short predicted half-lives, consistent with rapid systemic elimination.

Toxicity: Predicted probabilities for hERG channel inhibition were low for both compounds, suggesting minimal cardiotoxic risk. Other toxicity endpoints also showed low predicted probabilities. However, antirrhinin displayed slightly higher predicted values in several toxicity-related parameters compared with primulin.

In summary, the predicted ADMET profiles indicate that both anthocyanins possess limited oral bioavailability and low blood–brain barrier permeability. Primulin demonstrated a comparatively more favorable pharmacokinetic profile, characterized by lower plasma protein binding and higher predicted clearance. Antirrhinin exhibited stronger plasma protein binding and slightly elevated toxicity-related probabilities.

**Figure 1:**
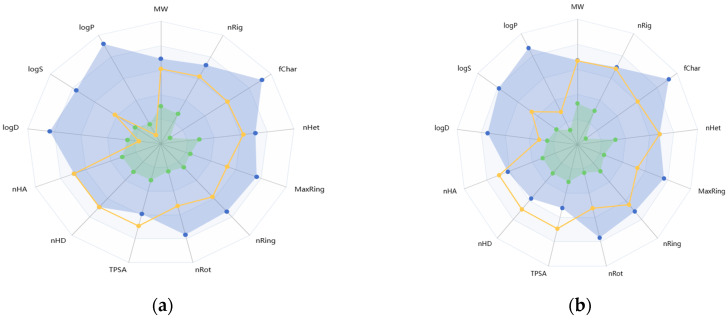
**Radial diagrams representing predicted ADMET properties of primulin (a) and antirrhinin (b).** The diagrams summarize key pharmacokinetic and toxicity parameters generated using ADMETlab 3.0. Color coding indicates the acceptable ranges of parameter values, while the connecting lines form an integrated ADMET profile, enabling clear visual comparison.

For visualization of the ADMET property distribution, the predicted parameters are also presented as radial diagrams (Figure 1). These diagrams illustrate the relative differences between the two compounds, highlighting the higher clearance and distribution parameters predicted for primulin and the stronger protein binding observed for antirrhinin.

Overall, the computational analysis indicates that primulin and antirrhinin exhibit distinct pharmacokinetic characteristics. Primulin shows a more favorable predicted pharmacokinetic profile, whereas antirrhinin demonstrates stronger plasma protein binding and slightly higher predicted toxicity probabilities. These differences may influence their potential biological activity and warrant further experimental investigation.

### 2.4. Molecular Dynamics Simulation

To evaluate the stability of the anthocyanin–cytokine complexes, molecular dynamics (MD) simulations were performed and several structural parameters were analyzed, including root-mean-square deviation (RMSD), radius of gyration (Rg), number of hydrogen bonds (H-bonds), solvent-accessible surface area (SASA), and root-mean-square fluctuation (RMSF). These parameters provide complementary information on protein conformational stability and ligand–protein interactions under conditions approximating a physiological environment.

**Figure 2:**
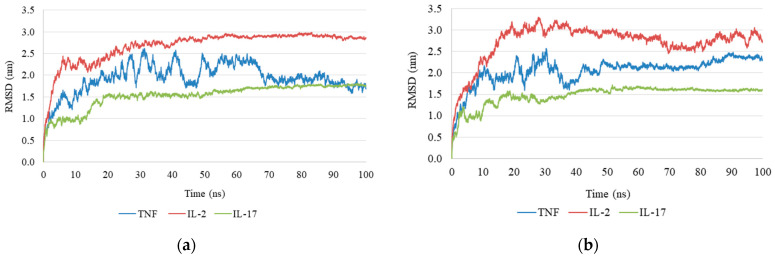
Root-mean-square deviation (RMSD) profiles of cytokine Cα atoms during molecular dynamics simulations: (**a**) cytokine complex with antirrhinin; (**b**) cytokine complex with primulin.

RMSD analysis indicated that the antirrhinin complex reached structural equilibrium within the first 10 ns of simulation and remained stable throughout the remaining trajectory (Figure 2a). In contrast, the primulin complex exhibited larger fluctuations over time, suggesting reduced structural stability of the ligand–protein complex (Figure 2b).

**Figure 3:**
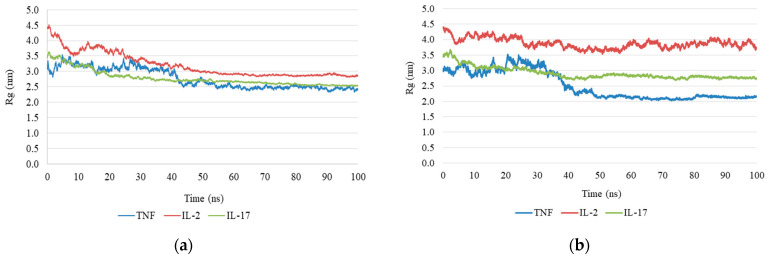
Radius of gyration (Rg) of cytokine–anthocyanin complexes during molecular dynamics simulations: (**a**) antirrhinin complex; (**b**) primulin complex.

Radius of Gyration: Analysis of the radius of gyration supported these observations. In the antirrhinin complex, the Rg value remained relatively constant during the simulation, indicating preservation of protein compactness (Figure 3a). In contrast, the primulin complex showed more pronounced fluctuations in Rg, suggesting partial loss of structural compactness (Figure 3b).

**Figure 4:**
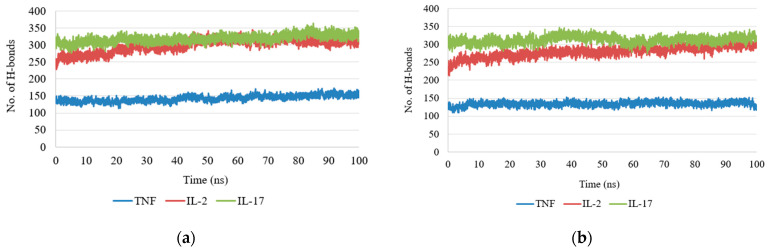
Number of hydrogen bonds observed in cytokine–anthocyanin complexes during molecular dynamics simulations: (**a**) antirrhinin complex; (**b**) primulin complex.

Hydrogen Bond Analysis: Hydrogen bond dynamics provided an additional measure of interaction stability. In the antirrhinin complex, the number of hydrogen bonds remained within a range of two to four throughout the simulation, indicating stable ligand retention within the binding region. In the primulin complex, the number of hydrogen bonds decreased during later stages of the simulation, suggesting weaker or less persistent interactions (Figure 4).

**Figure 5:**
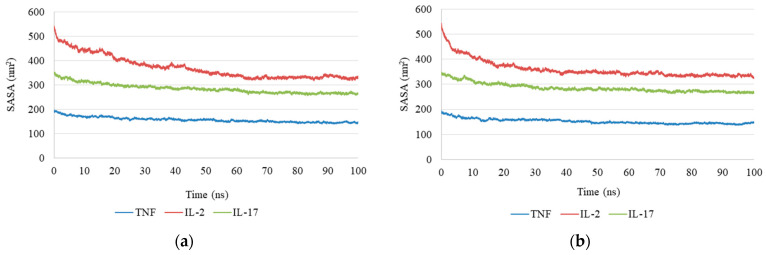
Solvent-accessible surface area (SASA) of cytokine–anthocyanin complexes during molecular dynamics simulations: (**a**) antirrhinin complex; (**b**) primulin complex.

SASA Solvent-Accessible Surface Area: SASA analysis revealed a gradual decrease in solvent-accessible surface area for the antirrhinin complex during the first 30 ns of simulation (from approximately 500 to ~350 Å^2^), after which the values stabilized (Figure 5a). This behavior indicates increased protein compactness and stabilization of the complex. A decrease in SASA was also observed for the primulin complex, although the change was less pronounced (approximately 300–330 Å^2^), reflecting greater mobility of the protein surface (Figure 5b).

The solvent-accessible surface area (SASA) of the protein–ligand complexes exhibited a gradual decrease over the course of the simulation. To elucidate the origin of this trend, both global structural parameters and local conformational changes were analyzed. The relatively stable root mean square deviation (RMSD) and radius of gyration (Rg) profiles indicate that the overall protein structure remained compact and did not undergo global collapse. Instead, the observed reduction in SASA is primarily attributed to local rearrangements within the binding site. In particular, residues forming the hydrophobic pocket showed decreased solvent exposure, suggesting a ligand-induced pocket closure and tighter packing around the ligand. This behavior reflects stabilization of the protein–ligand complex through enhanced hydrophobic interactions rather than large-scale conformational changes in the protein.

**Figure 6:**
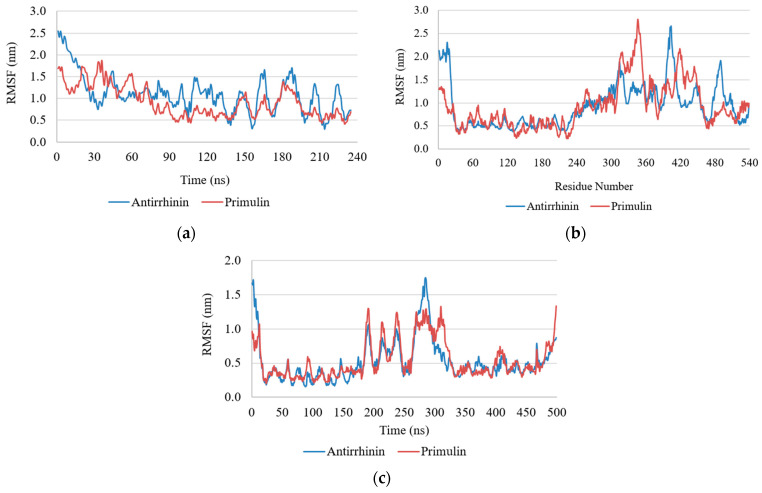
Root-mean-square fluctuation (RMSF) profiles of cytokine residues during molecular dynamics simulations with anthocyanins: (**a**) TNF; (**b**) IL-2; (**c**) IL-17.

RMSF Analysis: Root-mean-square fluctuation (RMSF) analysis revealed differences in protein flexibility depending on both the cytokine and the bound ligand. In the case of IL-2, the highest mobility was observed in the N- and C-terminal regions, where RMSF values exceeded 2.5 nm, whereas the central portion of the protein remained relatively stable (<1 nm), with several localized peaks indicating increased flexibility (Figure 6).

IL-17 exhibited a more compact and stable structure, with RMSF values generally below 1 nm across most residues and only minor fluctuations at the terminal regions. By contrast, TNF showed greater structural mobility, with RMSF values reaching approximately 1.5 nm in regions near the N-terminal portion of the protein.

Comparison of ligand-bound complexes indicated that primulin reduced fluctuation amplitudes in TNF and IL-17 relative to antirrhinin. For IL-2, however, both ligands produced similar RMSF profiles, including elevated fluctuations in the terminal regions.

Combined analysis of RMSD, Rg, hydrogen bonding, SASA, and RMSF indicates that antirrhinin–cytokine complexes exhibit greater structural stability than primulin–cytokine complexes. Antirrhinin binding was associated with increased protein compactness, more persistent hydrogen bonding, and reduced solvent exposure. In contrast, primulin complexes showed larger fluctuations and less stable hydrogen-bond interactions.

These results indicate that antirrhinin (cyanidin-3-O-rutinoside chloride) forms more stable complexes with the analyzed cytokines, whereas primulin (malvidin-3-galactoside) exhibits moderate stability while maintaining detectable ligand–protein interactions.

**Figure 7:**
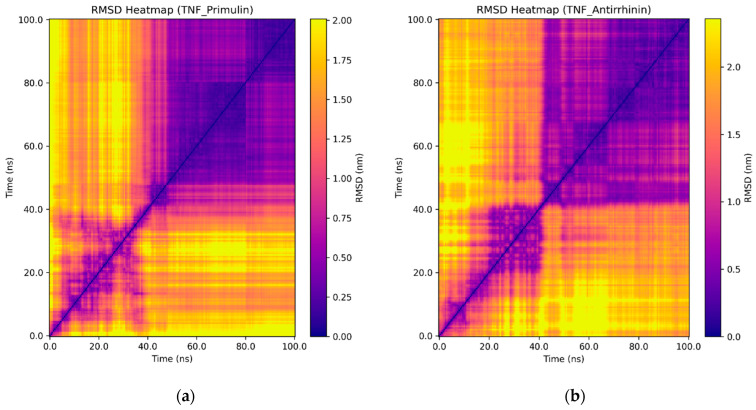

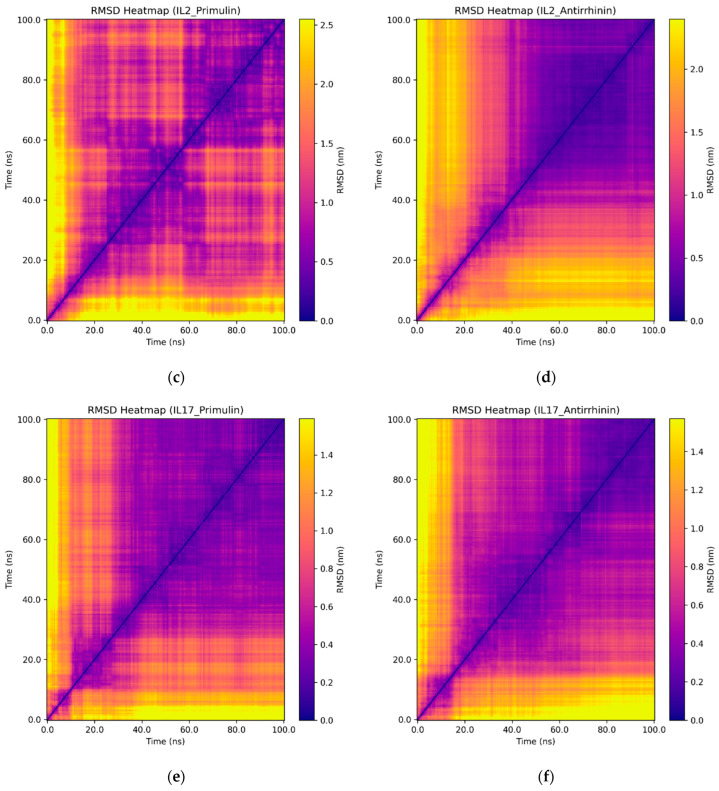
RMSD heatmaps illustrating conformational changes in cytokines during molecular dynamics simulations with anthocyanins: TNF ((**a**), primulin; (**b**), antirrhinin), IL-2 ((**c**), primulin; (**d**), antirrhinin), and IL-17 ((**e**), primulin; (**f**), antirrhinin).

Additional insights into the dynamics of the complexes were provided by RMSD heatmaps, which allowed tracking of conformational changes in the proteins throughout the simulations (Figure 7).

For the TNF–primulin complex, multiple regions with high RMSD values (yellow zones) were observed, indicating instability and frequent conformational transitions. In contrast, the TNF–antirrhinin complex was characterized by a more homogeneous map with a predominance of dark violet areas, particularly in the second half of the simulation, reflecting structural stabilization.

In the case of IL-2, the primulin complex showed substantial fluctuations throughout the simulation, as evidenced by heterogeneous regions on the map. Conversely, the IL-2–antirrhinin complex maintained a more stable conformation, confirmed by the prevalence of low RMSD values.

For IL-17, the maps of both complexes appeared relatively stable; however, in the presence of antirrhinin, fewer high RMSD zones were detected compared to primulin.

Thus, the RMSD heatmap results are consistent with the analyses of RMSD, Rg, H-bonds, SASA, and RMSF, confirming that antirrhinin–cytokine complexes exhibit higher structural stability compared to primulin–cytokine complexes.

Overall, the results of molecular docking and dynamic stability analysis indicate that the anthocyanins primulin and antirrhinin can effectively interact with key cytokine targets (TNF, IL-2, IL-17), altering their structural flexibility and potential biological activity. The in silico findings are supported by in vivo experiments, where these compounds significantly modulated cytokine gene expression. Taken together, the data suggest that the identified differences in molecular binding mechanisms are reflected in the distinct immunomodulatory effects observed at the cellular response level.

### 2.5. Gene Expression

In the in vivo experiment, BALB/c mice were administered the anthocyanins primulin and antirrhinin. During the three-day observation period, no signs of toxicity, inflammatory reactions, or behavioral abnormalities were detected, indicating good tolerability of the tested compounds.

Seventy-two hours after administration, peritoneal macrophages were isolated for molecular analysis. Total RNA extracted from these cells showed acceptable quality parameters (concentration 80–150 ng/µL; A260/A280 ≈ 1.9–2.0). Complementary DNA (cDNA) was synthesized from the RNA samples and used for quantitative real-time PCR (qPCR) analysis.

**Figure 8:**
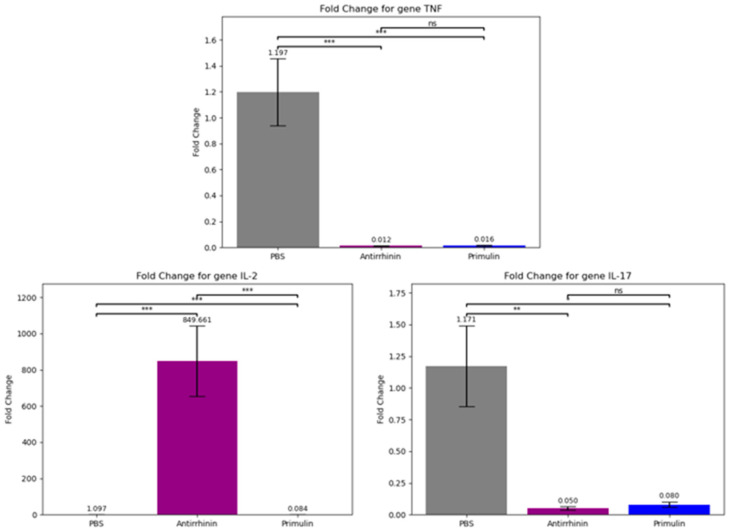
**Relative expression of TNF, IL-2, and IL-17 genes in peritoneal macrophages isolated from BALB/c mice following administration of primulin or antirrhinin.** Student’s *t*-test determined statistical significance: * *p* < 0.05, ** *p* < 0.01, *** *p* < 0.001; ns—not significant (*p* ≥ 0.05) Gene expression was quantified by qPCR and normalized to the reference gene A. Values represent mean ± SD (*p* < 0.05 vs. control).

Expression levels of the cytokine genes TNF, IL-2, and IL-17 were evaluated relative to the reference gene Actb. Compared with the control group (PBS), both anthocyanins significantly reduced TNF expression, indicating suppression of pro-inflammatory signaling. However, the compounds differed in their effects on IL-2 expression. Antirrhinin markedly increased IL-2 transcription, whereas primulin produced a reduction in IL-2 expression, indicating distinct immunomodulatory activities (Figure 8).

The qPCR results were statistically analyzed using Student’s *t*-test for pairwise comparisons and one-way ANOVA to assess differences across all groups. For all analyzed genes, the differences were statistically significant (*p* < 0.05), confirming the reliability of the observed effects.

The data demonstrate the potential of anthocyanins as regulators of inflammatory cytokine expression. Primulin exhibited a pronounced suppressive effect on TNF and IL-17. Conversely, antirrhinin not only inhibited pro-inflammatory markers but also induced IL-2 expression, suggesting a potential role in activating T-cell responses.

Consequently, primulin and antirrhinin extracts display potent but distinct immunomodulatory profiles. While primulin primarily exerted an anti-inflammatory action by reducing the expression of all three cytokines, antirrhinin simultaneously suppressed inflammation (TNF, IL-17) and enhanced IL-2 production, indicating its capacity to stimulate cellular immune responses.

Significantly, these in vivo results correlate closely with the in silico findings. Molecular docking and molecular dynamics simulations revealed differences in the stability of primulin– and antirrhinin–cytokine complexes, which were reflected at the cellular level. Antirrhinin, which exhibited more stable binding with IL-2 and IL-17, successfully induced IL-2 expression and reduced pro-inflammatory levels in the experiment. In contrast, primulin demonstrated lower binding stability and predominantly suppressed the expression of all investigated genes.

These findings confirm that combining in silico and in vivo approaches provides a comprehensive evaluation of the immunomodulatory potential of natural anthocyanins, facilitating the identification of compounds with promising biomedical properties.

## 3. Discussion

Anthocyanins represent an important group of plant-derived bioactive compounds with diverse biological activities and potential applications in functional nutrition and disease prevention [22]. These water-soluble flavonoid pigments produce coloration ranging from red and purple to blue, depending on molecular structure and environmental pH. Increasing experimental, clinical, and epidemiological evidence indicates that anthocyanins influence pathological processes associated with cancer, metabolic syndrome, cardiovascular disorders, and neurodegenerative diseases [23]. At the molecular level, anthocyanins interact with regulatory proteins and components of intracellular signaling pathways, thereby affecting gene expression, inflammatory responses, and cellular homeostasis [24].

This study investigated the biological activity of two structurally related anthocyanins, primulin and antirrhinin, using an integrated experimental and computational framework. The analysis combined molecular docking, molecular dynamics simulations, ADMET prediction, and experimental evaluation of cytokine gene expression in immunocompetent cells to characterize their potential immunomodulatory properties.

ADMET predictions indicated that both compounds display moderate pharmacokinetic properties. Their relatively high polarity, reflected by a topological polar surface area (TPSA) exceeding 180 Å^2^, is consistent with the predicted low intestinal permeability in the Caco-2 model, although the calculated human intestinal absorption index remained relatively high (HIA > 0.85). Both anthocyanins were predicted to act as substrates of P-glycoprotein (P-gp), indicating that active efflux transport may reduce their systemic bioavailability. Neither compound showed inhibitory activity toward major cytochrome P450 isoenzymes, suggesting a low potential for metabolic drug–drug interactions and supporting the generally favorable safety profile associated with dietary anthocyanins [25].

Molecular docking analysis revealed high binding affinities of both anthocyanins for the target cytokines. The calculated binding energies (antirrhinin: from −8.4 to −9.6 kcal/mol for TNF-α, from −9.5 to −8.6 kcal/mol for IL-2, and from −11.6 to −10.0 kcal/mol for IL-17; primulin: from −7.3 to −6.4 kcal/mol for TNF-α, from −8.1 to −7.1 kcal/mol for IL-2, and from −9.2 to −8.6 kcal/mol for IL-17) suggest the formation of thermodynamically stable ligand–protein complexes stabilized by hydrogen bonds, π–π stacking, and hydrophobic interactions. Molecular dynamics simulations performed over 100 ns confirmed the stability of these complexes. The root-mean-square deviation (RMSD) values for the anthocyanins and IL-17 remained below 20.0 Å (2.0 nm) throughout the simulation trajectory. For IL-2, the complex with antirrhinin exhibited RMSD values not exceeding 30.0 Å (3.0 nm), with a stable trajectory observed after 50 ns. In contrast, the complex with primulin was less stable: RMSD values exceeded 33.0 Å (3.3 nm) at 30 ns, followed by a decrease without convergence to a stable trajectory. The RMSD profiles for TNF-α showed the opposite trend. The antirrhinin complex displayed greater flexibility, with RMSD fluctuations between 15.0 Å (1.5 nm) and 26.0 Å (2.6 nm), eventually stabilizing within the range of 17.0 Å (1.7 nm) to 22.0 Å (2.2 nm) before decreasing. The primulin complex demonstrated more stable behavior for TNF-α, with RMSD values ranging from 21.0 Å (2.1 nm) to 24.0 Å (2.4 nm) after 50 ns. The preservation of key intermolecular contacts indicates that both ligands maintain stable binding conformations within the predicted interaction regions, particularly in the case of IL-17, despite their limited conformational flexibility.

Experimental evaluation of cytokine expression revealed distinct immunomodulatory profiles for the two anthocyanins. Both compounds significantly reduced the expression of the pro-inflammatory cytokines TNF and IL-17, consistent with previously reported anti-inflammatory activities of anthocyanins [26,27]. The two compounds differed, however, in their effects on IL-2 expression. Antirrhinin markedly increased IL-2 transcription, whereas primulin suppressed IL-2 expression. Given the central role of IL-2 in T-cell activation, proliferation, and immune homeostasis, these opposing effects may have important functional implications. The stimulation of IL-2 expression by antirrhinin suggests a capacity to enhance adaptive immune responses under conditions of immune suppression or impaired cellular immunity. In contrast, the reduction in IL-2 expression by primulin indicates a more pronounced immunosuppressive profile, which could be beneficial in inflammatory or autoimmune disorders.

The computational predictions correspond well with the experimental observations. Molecular modeling indicated stronger and more stable interactions of antirrhinin with IL-2 and IL-17 compared with primulin. Consistent with these predictions, antirrhinin increased IL-2 expression while simultaneously suppressing pro-inflammatory cytokines in the cellular assays. Primulin formed comparatively less stable complexes in the simulations and showed predominantly inhibitory effects on cytokine expression. The combined computational and experimental results therefore provide complementary evidence supporting the distinct immunomodulatory behavior of the two compounds.

These observations are consistent with the broader literature describing anthocyanins as modulators of inflammatory signaling pathways. Previous studies have demonstrated that anthocyanins can reduce the production of pro-inflammatory mediators such as TNF-α and IL-1β through suppression of NF-κB and MAPK signaling cascades. Clinical studies further indicate that sustained dietary intake of anthocyanin-rich foods may decrease circulating concentrations of inflammatory cytokines, including TNF-α and IL-6, particularly in individuals with metabolic disorders [26,27].

Taken together, the results demonstrate that primulin and antirrhinin exhibit distinct immunomodulatory properties despite their structural similarity. Primulin primarily displays anti-inflammatory and immunosuppressive activity, whereas antirrhinin combines suppression of pro-inflammatory cytokines with stimulation of IL-2-mediated immune responses. These differences suggest potential therapeutic applications depending on the immunological context. Additional studies are required to clarify their mechanisms of action, including dose–response relationships, involvement of additional signaling pathways, and validation in extended in vivo models.

## 4. Materials and Methods

### 4.1. Materials

**Figure 9:**
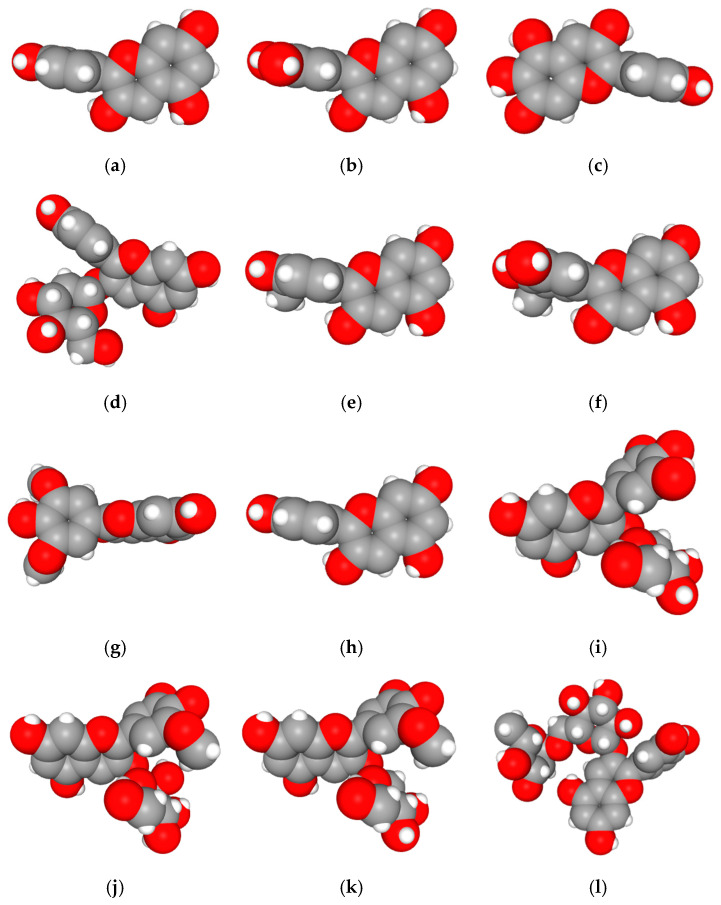
**Three-dimensional molecular structures of the investigated anthocyanins and anthocyanidins:** (**a**) Cyanidin, (**b**) Delphinidin, (**c**) Aurantinidin, (**d**) Chrysanthemin, (**e**) Peonidin, (**f**) Petunidin, (**g**) Malvidin, (**h**) Pelargonidin, (**i**) Myrtillin, (**j**) Primulin, (**k**) Oenin, (**l**) Antirrhinin.

Twelve anthocyanins and anthocyanidins were selected as ligands: cyanidin, delphinidin, aurantinidin, chrysanthemin, peonidin, petunidin, malvidin, pelargonidin, myrtillin, primulin, oenin, and antirrhinin (Figure 9) [28]. The two- and three-dimensional molecular structures of these compounds were obtained from the PubChem [29] and ChemSpider databases [30].

#### 4.1.1. Molecular Docking

Molecular docking was performed to evaluate the interactions between anthocyanins and key cytokines (TNF, IL-2, and IL-17) using AutoDock Vina version 1.2 and AutoDockTools [31,32]. Protein structures were obtained from the AlphaFold database [33]. Prior to docking, protein structures were prepared by removing water molecules, adding polar hydrogen atoms, assigning Gasteiger charges, and optimizing geometry using PyMOL (http://www.pymol.org/pymol) and Open Babel 3.1,1 [34,35,36]. Binding sites were defined based on previously reported functional regions of the proteins. Binding affinity was evaluated according to predicted binding energies and ligand orientations within the protein–ligand complexes. The docking grid dimensions and exhaustiveness parameters were optimized to cover the predicted binding regions of the cytokines.

To perform molecular docking, a semi-blind approach was employed to minimize bias associated with the a priori definition of the ligand-binding site. The search space was configured to encompass the largest possible surface area of the protein, thereby enabling the identification and analysis of multiple potential binding sites.

The docking parameters included fixed grid dimensions (size_x, size_y, size_z) of 40 × 40 × 40 Å, an energy range of 4 kcal/mol, and an exhaustiveness value of 8, representing a compromise between computational accuracy and efficiency.

#### 4.1.2. Interaction Analysis

Amino acid contacts and hydrogen bonding interactions were analyzed using LigPlot+ [37], revealing key residues that contribute to the stabilization of ligand–protein complexes.

#### 4.1.3. ADMET Analysis

The pharmacokinetic and toxicological properties of primulin and antirrhinin were predicted using the ADMETlab 3.0 platform [38]. The analysis included parameters related to absorption, distribution, metabolism, excretion, and toxicity (ADMET).

#### 4.1.4. Molecular Dynamics

The dynamic stability of the protein–ligand complexes was examined through 100 ns molecular dynamics (MD) simulations carried out in GROMACS. Protein topology was described by the AMBER99SB-ILDN force field; ligand parameters were derived within the General AMBER Force Field (GAFF) framework, with partial atomic charges assigned by the AM1-BCC method.

Each system was placed in a periodic cubic simulation box, maintaining a minimum solute-to-boundary distance of 10 Å. Explicit solvation was achieved with the TIP3P water model, and Na^+^/Cl^−^ ions were introduced to neutralize the total charge and to reproduce a physiological ionic strength of 0.15 M. Under near-physiological conditions (pH 7.4), protonation states of ionizable protein residues, including histidines, were assigned using PROPKA based on predicted pKa values. Anthocyanins were modeled in their quinonoidal base forms, which are considered the predominant species at neutral pH according to experimental and computational studies. The selected protonation state was used consistently during ligand parameterization within the GAFF framework, with AM1-BCC charges reflecting the corresponding electronic distribution.

Prior to dynamics, energy minimization was performed by the steepest descent algorithm and terminated when the maximum force dropped below 1000 kJ·mol^−1^·nm^−1^. The minimized systems were subsequently equilibrated in two sequential phases—1 ns under NVT conditions at 300 K (velocity-rescaling thermostat), followed by 1 ns under NPT conditions at 1 atm (Parrinello–Rahman barostat)—with harmonic position restraints applied to all non-hydrogen solute atoms throughout.

Production runs of 100 ns were conducted under periodic boundary conditions with a 2 fs integration step. Covalent bonds involving hydrogen atoms were constrained with the LINCS algorithm. Long-range electrostatics were handled by particle mesh Ewald (PME) summation with a real-space cutoff of 10 Å; van der Waals interactions were truncated at 10 Å using a force-switch function acting over the 8–10 Å range. Neighbor lists were rebuilt every 10 steps. Temperature and pressure were held at 300 K and 1 atm, respectively, with coupling time constants of 0.1 ps and 2.0 ps, and coordinates were recorded at 10 ps intervals.

Trajectory analyses relied on standard GROMACS utilities supplemented by additional tools. Global structural stability was assessed from the root-mean-square deviation (RMSD) and radius of gyration (Rg), while local backbone flexibility was characterized by per-residue root-mean-square fluctuation (RMSF) to delineate mobile and rigid segments of the protein.

Collective motions were examined by principal component analysis (PCA). The covariance matrix of atomic positional fluctuations was constructed after removal of overall translational and rotational motion and subsequently diagonalized; the first two eigenvectors (PC1 and PC2) were retained as descriptors of the dominant conformational dynamics.

The conformational free energy landscape (FEL) was constructed by projecting the MD trajectories onto the first two principal components (PC1 and PC2). The free energy was calculated using the Boltzmann inversion formula:

G = −kBT ln P,

where P is the probability distribution of conformational states, kB is the Boltzmann constant, and T is the temperature. This analysis enabled the identification of stable conformational basins and transition states.

Binding free energies were estimated by the Molecular Mechanics/Poisson–Boltzmann Surface Area (MM-PBSA) approach using snapshots extracted at uniform intervals from the final 20 ns of each trajectory. The binding free energy was decomposed as:

ΔG_bind = G_complex − (G_protein + G_ligand),

Each component comprises the molecular mechanics energy (electrostatic and van der Waals terms), the polar solvation contribution obtained by solving the Poisson–Boltzmann equation, and the nonpolar solvation term estimated from the solvent-accessible surface area (SASA).

#### 4.1.5. In Vivo Experiments

Following in silico validation, the immunomodulatory activity of the selected compounds was evaluated in male BALB/c mice (n = 12). The animals were randomly assigned into three groups (n = 4 per group): primulin-treated, antirrhinin-treated, and control (phosphate-buffered saline, PBS). The compounds were administered via intraperitoneal injection at a dose of 1 mg per animal.

After 72 h post-administration, peritoneal macrophages were harvested under sterile conditions and stored at −80 °C for subsequent molecular analyses [39].

All experimental procedures involving animals were conducted in accordance with ethical standards and were approved by the Institutional Animal Care and Use Committee (IACUC) of the Research and Production Center for Microbiology and Virology (protocol code: 02-09-184; approval date: 30 October 2023).

#### 4.1.6. Gene Expression Analysis

Total RNA was extracted from macrophages and reverse transcribed into cDNA. Gene expression levels were analyzed by quantitative PCR (qPCR) using a QuantStudio™ 5 system with TaqMan probes specific for IL-2, IL-17, TNF, and Actb (reference gene). Relative gene expression levels were calculated using the 2^−ΔΔCt^ method [23].

Data processing and graphical visualization were performed in Microsoft Excel. Statistical analysis was conducted using one-way analysis of variance (ANOVA), followed by Fisher’s least significant difference (LSD) post hoc test to compare experimental and control groups. Data are presented as mean ± standard deviation (SD), and differences were considered statistically significant at *p* < 0.05.

## 5. Conclusions

Antirrhinin and primulin exhibited potent anti-inflammatory activity, primarily through suppression of the pro-inflammatory cytokines TNF and IL-17. Despite their structural relatedness, the two compounds exerted divergent effects on IL-2 expression: antirrhinin upregulated IL-2, whereas primulin downregulated it, indicating target-specific immunomodulatory profiles that cannot be inferred solely from scaffold similarity.

These contrasting effects carry distinct therapeutic implications. The capacity of antirrhinin to enhance IL-2 expression positions it as a candidate for applications requiring augmented cellular immune responses, whereas the broader suppressive profile of primulin is more consistent with utility in chronic inflammatory conditions. The differential regulation of IL-2 further provides mechanistic insight into the functional divergence achievable within the anthocyanin class of natural compounds.

Taken together, the present findings support anthocyanins as a productive chemical class for immunomodulatory lead development and demonstrate that integrating structure-based computational modeling with targeted experimental validation constitutes an efficient strategy for mechanism-informed prioritization of natural product candidates. Subsequent work should address pathway-level mechanisms of action, establish dose–response relationships, and assess activity in disease-relevant in vivo models to advance the translational potential of these compounds [40,41,42,43,44].

## Figures and Tables

**Table 1:** **Predicted binding energies (kcal/mol) of anthocyanins and anthocyanidins docked with TNF-α, IL-2, and IL-17**. Maximum and minimum docking scores obtained from AutoDock Vina simulations are reported for each ligand–cytokine complex.

#	Compounds	Binding Energy (kcal/mol)					
		TNF-α		IL-2		IL-17	
		Max	Min	Max	Min	Max	Min
1. A	Cyanidin	−5.6	−5.1	−6.2	−5.5	−7.5	−6.6
2. B	Delphinidin	−5.6	−5.0	−6.1	−5.6	−7.4	−6.1
3. C	Aurantinidin	−5.6	−4.9	−6.8	−5.9	−7.6	−6.3
4. D	Chrysanthemin	−5.9	−5.5	−6.7	−6.2	−8.9	−7.5
5. E	Peonidin	−5.9	−5.0	−6.2	−5.6	−7.3	−6.8
6. F	Petunidin	−6.1	−5.3	−6.2	−5.9	−7.6	−6.7
7. G	Malvidin	−5.4	−5.0	−6.0	−5.7	−7.4	−6.8
8. H	Pelargonidin	−5.5	−4.8	−7.2	−5.8	−7.5	−6.5
9. I	Myrtillin	−6.9	−6.5	−7.5	−7.1	−9.8	−8.9
10. J	Primulin	−7.3	−6.4	−8.1	−7.1	−9.2	−8.6
11. K	Oenin	−5.7	−5.4	−6.0	−5.8	−8.5	−7.3
12. L	Antirrhinin	−9.6	−8.4	−9.5	−8.6	−11.6	−10.0

**Table 2:** **Key amino acid interactions of primulin and antirrhinin with TNF-α, IL-2, and IL-17 identified by LigPlot+ analysis.** Hydrogen bonds and hydrophobic contacts within the predicted ligand–protein binding sites are listed for each cytokine–anthocyanin complex.

**2D visualization of the interaction of primulin with TNF-α**	**H-bonds**	**Hydrophobic interactions**
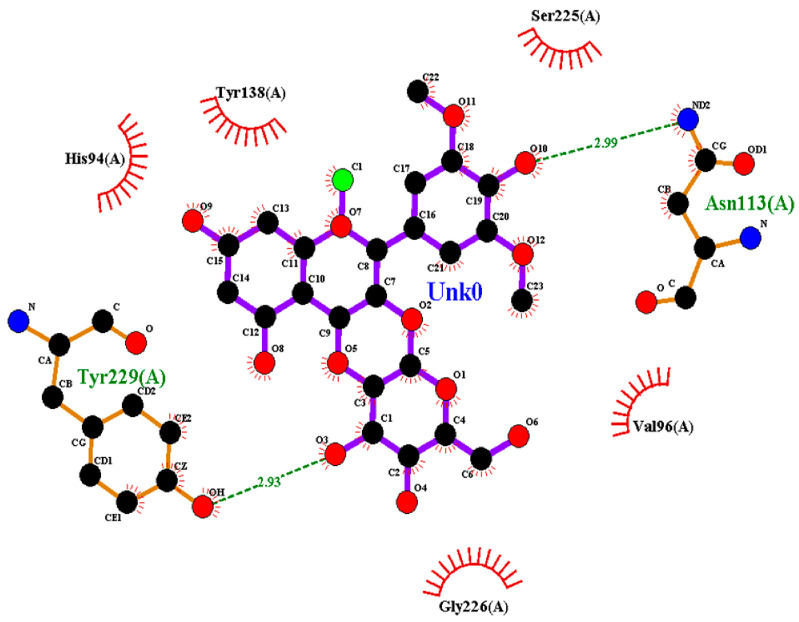	Asn113(A), Tyr229(A)	Ser225(A), Tyr138(A), His94(A), Val96(A), Gly226(A)
**2D visualization of the interaction of antirrhinin with TNF-α**	**H-bonds**	**Hydrophobic interactions**
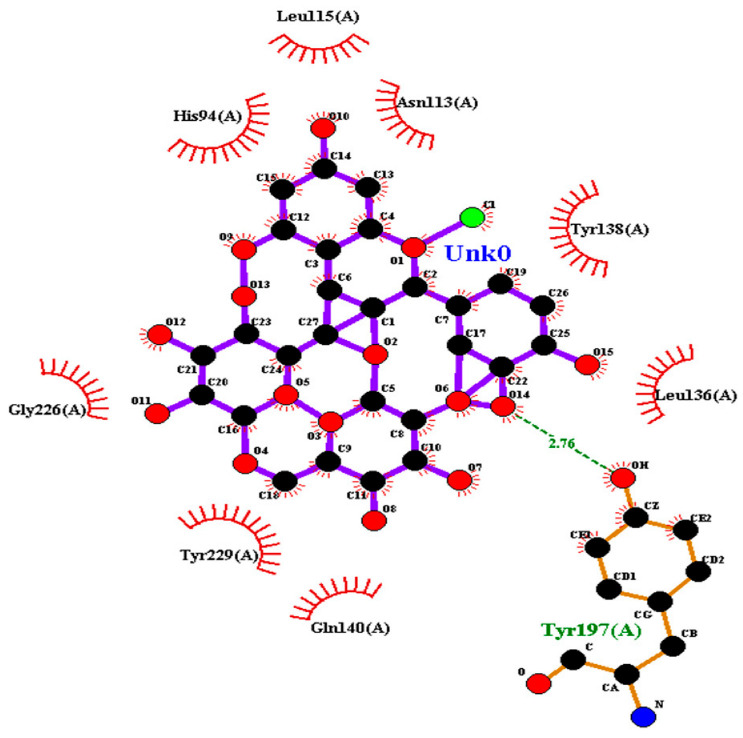	Tyr197(A)	His94(A), Leu115(A), Asn113(A), Tyr138(A), Leu136(A), Gly226(A), Tyr229(A), Gln140(A)
**2D visualization of the interaction of primulin with IL-2**	**H-bonds**	**Hydrophobic interactions**
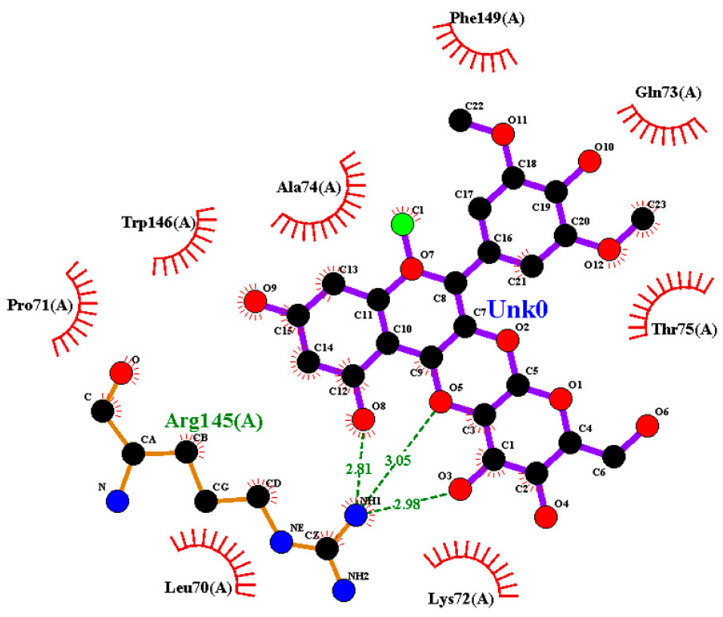	Arg145(A)	Pro71(A), Trp146(A), Ala74(A), Phe149(A), Gln73(A), Thr75(A), Leu70(A), Lys72(A)
**2D visualization of the interaction of antirrhinin with IL-2**	**H-bonds**	**Hydrophobic interactions**
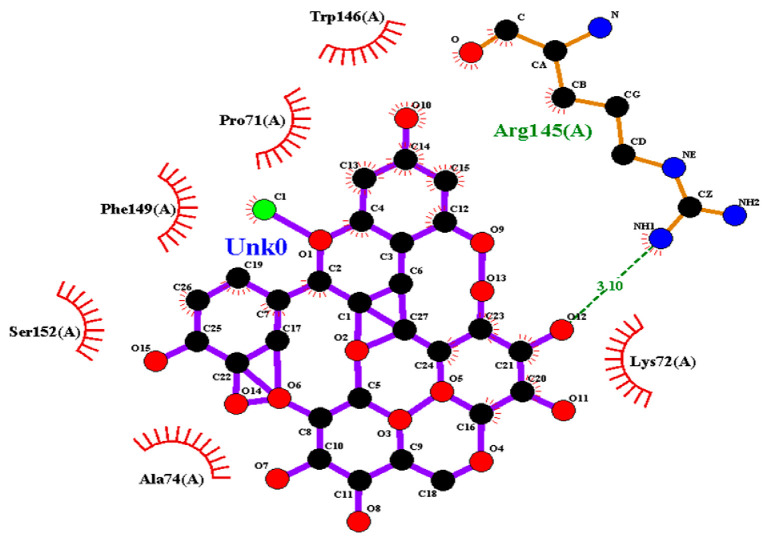		Trp146(A), Pro71(A), Phe149(A), Ser152(A), Ala74(A), Lys72(A)
**2D visualization of the interaction of primulin with IL-17**	**H-bonds**	**Hydrophobic interactions**
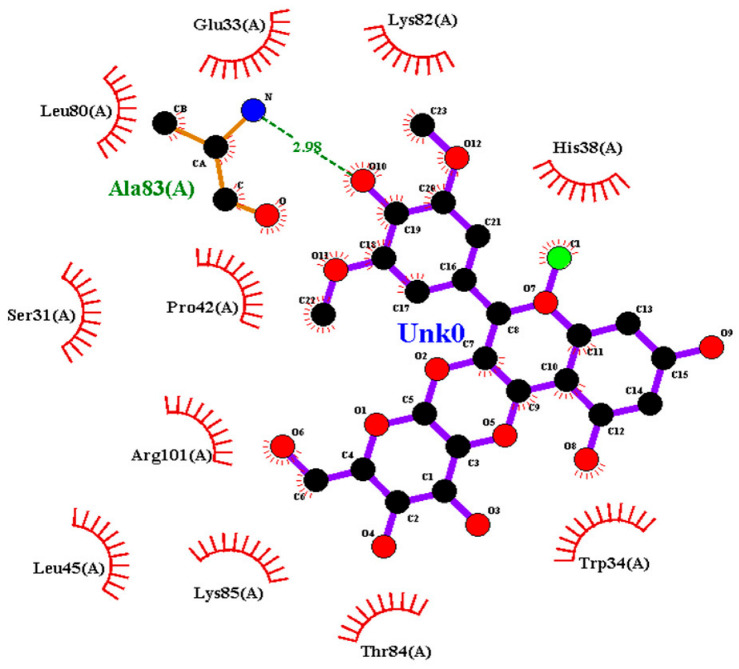	Ala83(A)	Leu80(A), Glu33(A), Lys82(A), His38(A), Ser31(A), Pro42(A), Arg101(A), Leu45(A), Lys85(A), Thr84(A), Trp34(A)
**2D visualization of the interaction of antirrhinin with IL-17**	**H-bonds**	**Hydrophobic interactions**
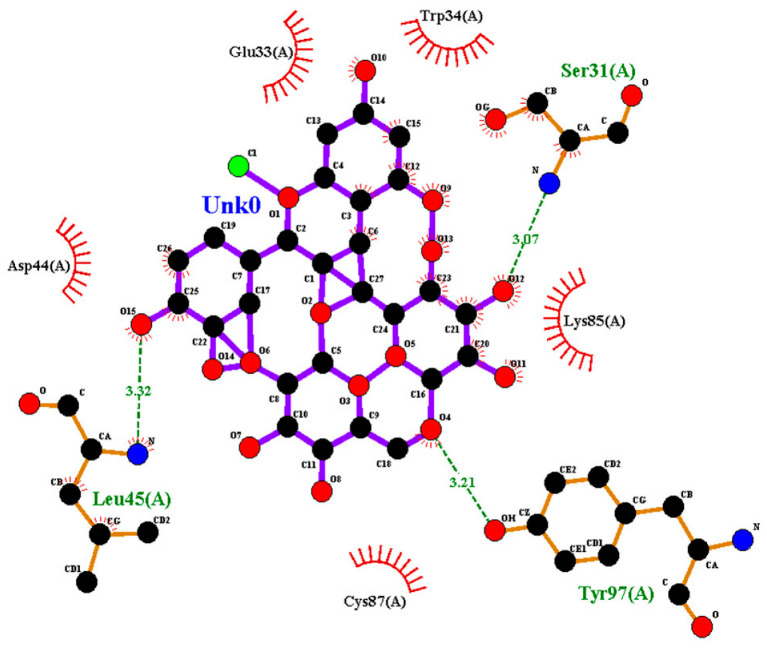	Ser31(A), Leu45(A), Tyr97(A)	Trp34(A), Glu33(A), Asp44(A), Lys85(A), Cys87(A)

**Table 3:** **Predicted ADMET parameters of primulin and antirrhinin obtained using the ADMETlab 3.0 platform.** Values represent predicted pharmacokinetic and toxicological properties, including absorption, distribution, metabolism, excretion, and toxicity endpoints.

Property	Model Name	Unit	Primulin	Antirrhinin
Absorption	Caco-2 Permeability	Numeric (log Papp in 10^−6^ cm/s)	−6.221	−6.593
	MDCK Permeability	Numeric (log Papp in 10^−6^ cm/s)	−5.328	−5.02
	Pgp-inhibitor	Probability (0–1)	0.0	0.0
	Pgp-substrate	Probability (0–1)	0.831	0.827
	HIA	Probability (0–1)	0.818	0.746
	F_20%_	Probability (0–1)	0.478	0.984
	F_30%_	Probability (0–1)	0.981	1.0
Distribution	PPB	%	77.773	83.4
	BBB permeability	Numeric (log BB)	0.0	0.001
Metabolism	CYP1A2 inhibitor	Probability (0–1)	0.0	0.0
	CYP1A2 substrate	Probability (0–1)	0.0	0.0
	CYP2C19 inhibitor	Probability (0–1)	0.0	0.0
	CYP2C19 substrate	Probability (0–1)	0.002	0.0
	CYP2C9 inhibitor	Probability (0–1)	0.0	0.0
	CYP2C9 substrate	Probability (0–1)	0.999	0.009
	CYP2D6 inhibitor	Probability (0–1)	0.0	0.0
	CYP2D6 substrate	Probability (0–1)	0.995	0.0
	CYP3A4 inhibitor	Probability (0–1)	0.0	0.0
	CYP3A4 substrate	Probability (0–1)	0.009	0.0
Excretion	CL	Numeric (mL/min/kg)	2.483	1.495
	T_1/2_	Numeric (h)	3.121	5.143
Toxicity	hERG Blockers	Probability (0–1)	0.011	0.003
	H-HT	Probability (0–1)	0.001	0.002
Tox21 pathway	NR-PPAR-gamma	Probability (0–1)	0.0	0.001
	SR-ARE	Probability (0–1)	0.341	0.204
	SR-p53	Probability (0–1)	0.107	0.146

## Data Availability

All the data generated or analyzed during this study are included in the published article.

## References

[1] Zheng X., Zhang X., Zeng F. (2025). Biological functions and health benefits of flavonoids in fruits and vegetables: A contemporary review. Foods.

[2] Pietta P.G. (2000). Flavonoids as antioxidants. J. Nat. Prod..

[3] Hwang J.-W., Kim E.-K., Lee S.-J., Kim Y.-S., Moon S.-H., Jeon B.-T., Sung S.-H., Kim E.-T., Park P.-J. (2012). Antioxidant activity and protective effect of anthocyanin oligomers on H_2_O_2_-triggered G_2_/M arrest in retinal cells. J. Agric. Food Chem..

[4] Sadowska-Bartosz I., Bartosz G. (2024). Antioxidant activity of anthocyanins and anthocyanidins: A critical review. Int. J. Mol. Sci..

[5] Gilani S.J., Bin-Jumah M.N., Al-Abbasi F.A., Nadeem M.S., Imam S.S., Alshehri S., Ghoneim M.M., Afzal M., Alzarea S.I., Sayyed N. (2022). Rosinidin flavonoid ameliorates hyperglycemia, lipid pathways and proinflammatory cytokines in streptozotocin-induced diabetic rats. Pharmaceutics.

[6] Rathee P., Chaudhary H., Rathee S., Rathee D., Kumar V., Kohli K. (2009). Mechanism of action of flavonoids as anti-inflammatory agents: A review. Inflamm. Allergy-Drug Targets.

[7] Testai L., Martelli A., Cristofaro M., Breschi M.C., Calderone V. (2013). Cardioprotective effects of different flavonoids against myocardial ischaemia/reperfusion injury in Langendorff-perfused rat hearts. J. Pharm. Pharmacol..

[8] Bjune K., Halvorsen P.S., Wangensteen H., Leren T.P., Bogsrud M.P., Strøm T.B. (2024). Flavonoids regulate LDLR through different mechanisms tied to their specific structures. J. Lipid Res..

[9] Vinayagam R., Xu B. (2015). Antidiabetic properties of dietary flavonoids: A cellular mechanism review. Nutr. Metab..

[10] García-Lafuente A., Guillamón E., Villares A., Rostagno M.A., Martínez J.A. (2009). Flavonoids as anti-inflammatory agents: Implications in cancer and cardiovascular disease. Inflamm. Res..

[11] Yudina R.S., Gordeeva E.I., Shoeva O.Y., Tikhonova M.A., Khlestkina E.K. (2021). Anthocyanins as components of functional nutrition. Vavilov J. Genet. Breed..

[12] Les F., Cásedas G., Gómez C., Moliner C., Valero M.S., López V. (2021). The role of anthocyanins as antidiabetic agents: From molecular mechanisms to in vivo and human studies. J. Physiol. Biochem..

[13] Mao T., Akshit F.N.U., Mohan M.S. (2023). Effects of anthocyanin supplementation in diet on glycemic and related cardiovascular biomarkers in patients with type 2 diabetes: A systematic review and meta-analysis of randomized controlled trials. Front. Nutr..

[14] Strathearn K.E., Yousef G.G., Grace M.H., Roy S.L., Tambe M.A., Ferruzzi M.G., Wu Q.-L., Simon J.E., Lila M.A., Rochet J.-C. (2014). Neuroprotective effects of anthocyanin- and proanthocyanidin-rich extracts in cellular models of Parkinson’s disease. Brain Res..

[15] Beydoun M.A., Beydoun H.A., Wang Y. (2022). Long-term dietary flavonoid intake and risk of Alzheimer disease and related dementias among US adults. Am. J. Clin. Nutr..

[16] Monteiro A.F., Scotti L., Viana J.d.O., Nayarisseri A., Zondegoumba E.N., Junior F.J.B.M., Scotti M.T. (2018). Computational studies applied to flavonoids against Alzheimer’s and Parkinson’s diseases. Oxidative Med. Cell. Longev..

[17] Alshehri S., Imam S.S. (2021). Rosinidin attenuates lipopolysaccharide-induced memory impairment in rats: Possible mechanisms of action include antioxidant and anti-inflammatory effects. Biomolecules.

[18] Sravani M., Kumaran A., Dhamdhere A.T., Kumar N.S. (2021). Computational molecular docking analysis and visualisation of anthocyanins for anticancer activity. Int. J. Res. Appl. Sci. Biotechnol..

[19] Karcheva-Bahchevanska D., Nikolova M., Iliev I. (2023). Inhibitory potential of different bilberry (*Vaccinium myrtillus* L.) extracts on human salivary α-amylase. Molecules.

[20] Huang W.-Y., Liu Y.-M., Wang J., Wang X.-N., Li C.Y. (2014). Anti-inflammatory effect of the blueberry anthocyanins malvidin-3-glucoside and malvidin-3-galactoside in endothelial cells. Molecules.

[21] Thilavech T., Adisakwattana S. (2019). Cyanidin-3-rutinoside acts as a natural inhibitor of intestinal lipid digestion and absorption. BMC Complement. Med. Ther..

[22] Calderaro A., Barreca D., Bellocco E., Smeriglio A., Trombetta D., Laganà G., Nabavi S.M., Suntar I., Barreca D., Khan H. (2020). Colored phytonutrients: Role and applications in the functional foods of anthocyanins. Phytonutrients in Food: From Traditional to Rational Usage.

[23] Livak K.J., Schmittgen T.D. (2001). Analysis of relative gene expression data using real-time quantitative PCR and the 2^−ΔΔCT^ method. Methods.

[24] Li D., Wang P., Luo Y., Zhao M., Chen F. (2017). Health benefits of anthocyanins and molecular mechanisms: Update from recent decade. Crit. Rev. Food Sci. Nutr..

[25] Promyos N., Temviriyanukul P., Suttisansanee U. (2020). Investigation of Anthocyanidins and Anthocyanins for Targeting α-Glucosidase in Diabetes Mellitus. Prev. Nutr. Food Sci..

[26] Young A., Ajaz M., Vugic L., Shilton N. (2026). Anthocyanin Supplementation and Inflammation: A Systematic Review and Meta-Analysis of IL-8, IL-10, IL-18, IFN-γ, and Resistin in Healthy, Overweight, and Obese Populations. Food Sci. Nutr..

[27] Kozłowska A., Dzierżanowski T. (2021). Targeting Inflammation by Anthocyanins as the Novel Therapeutic Potential for Chronic Diseases: An Update. Molecules.

[28] Welch C.R., Wu Q., Simon J.E. (2008). Recent advances in anthocyanin analysis and characterization. Curr. Anal. Chem..

[29] Kim S., Chen J., Cheng T., Gindulyte A., He J., He S., Li Q., Shoemaker B.A., Thiessen P.A., Yu B. (2025). PubChem 2025 update. Nucleic Acids Res..

[30] Williams A.J. (2008). Public chemical compound databases. Curr. Opin. Drug Discov. Dev..

[31] AutoDock Vina. (n.d.). http://vina.scripps.edu.

[32] MGLTools—Molecular Graphics Laboratory. (n.d.). http://mgltools.scripps.edu/downloads.

[33] UniProt Consortium (2021). UniProt: The universal protein knowledgebase in 2021. Nucleic Acids Res..

[34] Tang S., Chen R., Lin M., Lin Q., Zhu Y., Ding J., Hu H., Ling M., Wu J. (2022). Accelerating AutoDock Vina with GPUs. Molecules.

[35] Schrödinger L., DeLano W. (2020). PyMOL. http://www.pymol.org/pymol.

[36] O’Boyle N.M., Banck M., James C.A., Morley C., Vandermeersch T., Hutchison G.R. (2011). Open Babel: An open chemical toolbox. J. Cheminform..

[37] Agrawal R., Punarva H.B., Heda G.O., Vishesh Y.M., Karunakar P. (2023). VinaLigGen: A method to generate LigPlots and retrieval of hydrogen and hydrophobic interactions from protein–ligand complexes. J. Biomol. Struct. Dyn..

[38] Fu L., Shi S., Yi J., Wang N., He Y., Wu Z., Peng J., Deng Y., Wang W., Wu C. (2024). ADMETlab 3.0: An updated comprehensive online ADMET prediction platform enhanced with broader coverage, improved performance, API functionality and decision support. Nucleic Acids Res..

[39] Law of the Republic of Kazakhstan No. 214-II of July 9, 2001. On State Support of Scientific and (or) Scientific and Technical Activities. https://adilet.zan.kz/rus/docs/V2000021512.

[40] Jiang X., Li X., Zhu C., Sun J., Tian L., Chen W., Bai W. (2018). The target cells of anthocyanins in metabolic syndrome. Crit. Rev. Food Sci. Nutr..

[41] Zhang X., Zhu Y., Song F., Yao Y., Ya F., Li D., Ling W., Yang Y. (2016). Effects of purified anthocyanin supplementation on platelet chemokines in hypocholesterolemic individuals: A randomized controlled trial. Nutr. Metab..

[42] Vendrame S., Klimis-Zacas D. (2015). Anti-inflammatory effect of anthocyanins via modulation of nuclear factor-κB and mitogen-activated protein kinase signaling cascades. Nutr. Rev..

[43] Sangsefidi Z.S., Hasanizadeh S., Hosseinzadeh M. (2018). Effect of purified anthocyanins or anthocyanin-rich extracts on C-reactive protein levels: A systematic review and meta-analysis of randomised clinical trials. Br. J. Nutr..

[44] Cenk E., Schmutz C., Pahlke G., Oertel A., Kollarova J., Mock H.-P., Matros A., Marko D. (2021). Immunomodulatory properties of blackberry anthocyanins in THP-1 derived macrophages. Int. J. Mol. Sci..

